# Rehabilitation potential in older people living with frailty: a systematic mapping review

**DOI:** 10.1186/s12877-021-02498-y

**Published:** 2021-10-07

**Authors:** Alison Cowley, Sarah E. Goldberg, Adam L. Gordon, Pip A. Logan

**Affiliations:** 1 Institute of Care Excellence, Derwent House, City Campus, Nottingham University Hospitals NHS Trust, Hucknall Road, Nottingham, NG5 1PB UK; 2 School of Medicine, University of Nottingham, Nottingham, UK; 3 School of Health Sciences, University of Nottingham, Nottingham, UK; 4 University Hospitals of Derby and Burton NHS Foundation Trust, Nottingham, UK; 5 NIHR Applied Research Collaboration East Midlands (ARC-EM), Nottingham, UK; 6 Nottingham CityCare Partnership CIC, Nottingham, UK

**Keywords:** Rehabilitation, Frail elderly, Geriatric assessment, Decision-making

## Abstract

**Background:**

Following periods of acute ill-health and injury, older people are frequently assessed and provided with rehabilitation services. Healthcare practitioners are required to make nuanced decisions about which patients are likely to benefit from and respond to rehabilitation. The clinical currency in which these decisions are transacted is through the term “rehabilitation potential”. The aim of this study was to explore information about rehabilitation potential in older people to inform the development of an evidence-based assessment tool.

**Methods:**

A systematic mapping review was completed to describe the extent of research and the concepts underpinning rehabilitation potential. We searched Medline, CINHAL, EMBASE, AMED, PsycINFO, PEDro, Cochrane Library, Web of Science, ProQuest, Trip and EThOS from inception to December 2020. We included studies which focused on rehabilitation potential and/or assessing for rehabilitation interventions for older people with comorbidities in the hospital and community setting. Reviewer pairs independently screened articles and extracted data against the inclusion criteria. A descriptive narrative approach to analysis was taken.

**Results:**

13,484 papers were identified and 49 included in the review. Rehabilitation potential was found to encompass two different but interrelated concepts of prognostication and outcome measurement. 1. Rehabilitation potential for prognostication involved the prediction of what could be achieved in programmes of rehabilitation. 2. Rehabilitation potential as an outcome measure retrospectively considered what had been achieved as a result of rehabilitation interventions. Assessments of rehabilitation potential included key domains which were largely assessed by members of the multi-disciplinary team at single time points. Limited evidence was identified which specifically considered rehabilitation potential amongst older people living with frailty.

**Conclusions:**

Current approaches to rehabilitation potential provide a snapshot of an individual’s abilities and conditions which fail to capture the dynamic nature and fluctuations associated with frailty and rehabilitation. New approaches to measures and abilities over time are required which allow for the prognostication of outcomes and potential benefits of rehabilitation interventions for older people living with frailty.

**Supplementary Information:**

The online version contains supplementary material available at 10.1186/s12877-021-02498-y.

## Background

Older people living with frailty often do not have discrete illnesses that they recover from. Rather they have an array of long-term conditions, which can both progressively worsen and have acute exacerbations resulting in hospitalisation. This can have a devastating impact on their function, well-being and social interactions.

Rehabilitation interventions are key in supporting patients’ recovery after periods of acute ill health [1, 2]. Healthcare practitioners are required to make nuanced decisions about patient’s rehabilitation requirements and which patients are likely to benefit from and respond to rehabilitation. The clinical currency in which these decisions are transacted is through the term “rehabilitation potential”.

Rehabilitation potential has been described in a number of different ways. It has been used to describe how well a patient’s function improves in response to rehabilitation, [3, 4] restoration of activities of daily living [5, 6] and patients’ psychological abilities to take part in rehabilitation [7]. Being deemed to have rehabilitation potential or not is critical to the amount and type of rehabilitation a patient will receive and can result in individuals being denied access to services which may be beneficial [8, 9]. How rehabilitation potential is conceptualised, assessed and operationalised, and which factors influence clinical decision-making, in routine clinical practice is highly variable.

The aim of our study was to identify and map literature on rehabilitation potential to inform the development of a tool to support consistent decisions [10, 11]. It sought to identify how the term rehabilitation potential or similar descriptors were used, what was understood by the term, how rehabilitation potential had been assessed, the use of clinical tools and decision-making frameworks, by whom they were used, and the timing of the assessment.

## Methods

We conducted a systematic mapping review. These are designed to describe the extent of research into a field and the concepts underpinning the research [12, 13]. They are widely used in developing complex interventions [14].

An electronic, three-step search strategy was used. An initial search was carried out in all databases using the keyword “rehabilitation potential”. A second search was carried out using MeSH combined with the key word “rehabilitation potential” across all included databases from inception to December 2020. Thirdly, a citation search was completed across the reference lists of all identified studies to enhance the rigour of the study [15]. Studies published in the English language were included. Databases searched were: Medline (Ovid 1946-present), CINAHL Plus with full text (EBSCO), EMBASE (Ovid), AMED (Allied and Complementary Medicine, Ovid), PsycINFO (Ovid), PEDro, Cochrane Library and Web of Science. The search for grey literature included: ProQuest Dissertations and Theses, Trip (Turning Research into Practice) and EThOS. Justification for the inclusion of each database can be found in supplementary data file one.

Searches, title and abstract screening were conducted by a single researcher (AC). Full text screening and data extraction were independently completed by two reviewers selected from AC, PL, SG and ALG. Disagreements were resolved through discussions with the study team. Data were recorded on a standardised data extraction form (supplementary data file two) which collected details about the study design, interventions, participants, context and outcomes alongside definitions of rehabilitation potential, methods of assessment and theoretical underpinnings. The form was piloted with a member of the study team on a sample of five papers to ensure that it was fit for purpose, unambiguous and clear.

### Inclusion criteria

Studies were included if they focused on rehabilitation interventions delivered in hospitals or community settings for adults aged over 65 with frailty or multiple comorbidities, where recovery trajectories are particularly uncertain. Studies that included assessments of rehabilitation potential and clinical decision-making during assessments for rehabilitation programmes were included. Studies which presented primary research, including randomized controlled trials, non-randomized controlled trials, quasi-experimental studies, before and after studies, prospective and retrospective cohort studies, case-control studies, analytical cross-sectional studies, case series, individual case reports, descriptive cross-sectional studies, phenomenology, grounded theory, ethnography and action research were included.

### Exclusion criteria

Studies focussing on specialist stroke rehabilitation, fracture care, end of life care or with a terminal diagnosis were excluded. Opinion pieces, editorials and books were excluded.

### Types of outcome

Outcomes of interest included measures of function or activities of daily living (ADL), instrumental activities of daily living (IADL), and access to and provision of services as a consequence of rehabilitation potential assessments.

### Data analysis

Data were analysed by publication rate by year, country of publication, study type, participant type and study settings. Results were displayed in descriptive tables taking into account a priori themes based on the World Health Organization International Classification of Functioning, Disability and Health (WHO ICF) [16] and emergent themes. This enabled the theoretical underpinnings and components of rehabilitation potential assessments relating to health conditions, body functions and structures, activities and participation personal and environmental factors to be identified and to inform the development of a rehabilitation potential assessment tool [10]. Categories were added into the analytical framework based upon important insights from included articles that were not adequately captured by a priori themes.

## Results

**Fig. 1:**
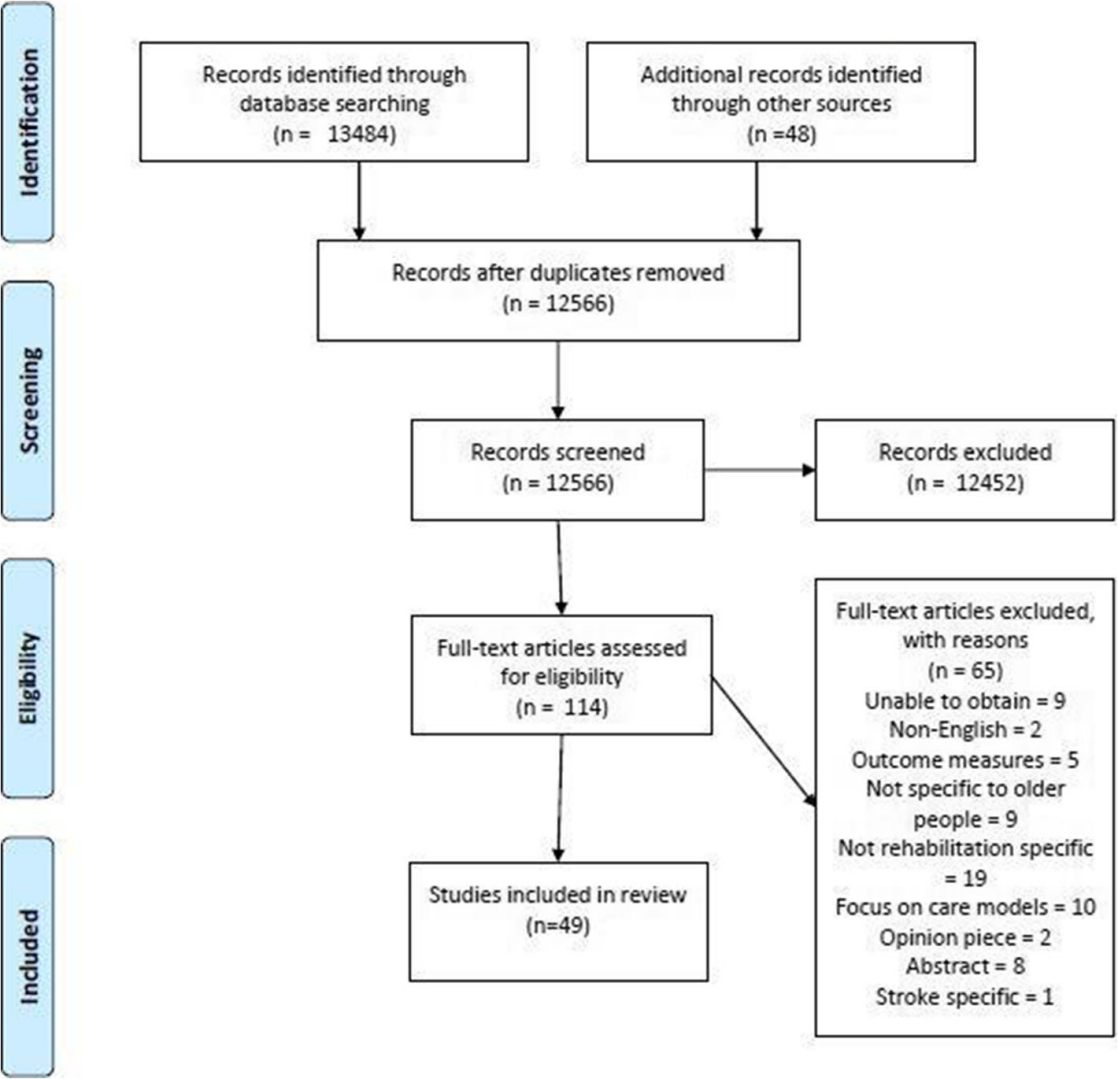
PRISMA diagram

13,484 papers were identified through bibliographic searches with an additional 48 found through citation searching. After duplicates were removed, 12,566 records titles and abstracts were screened and 12,452 were excluded. 114 articles underwent full paper screening, at which point a further 65 articles were excluded. 49 articles were included in the final review. A PRISMA diagram is shown in Fig. 1.

**Fig. 2:**
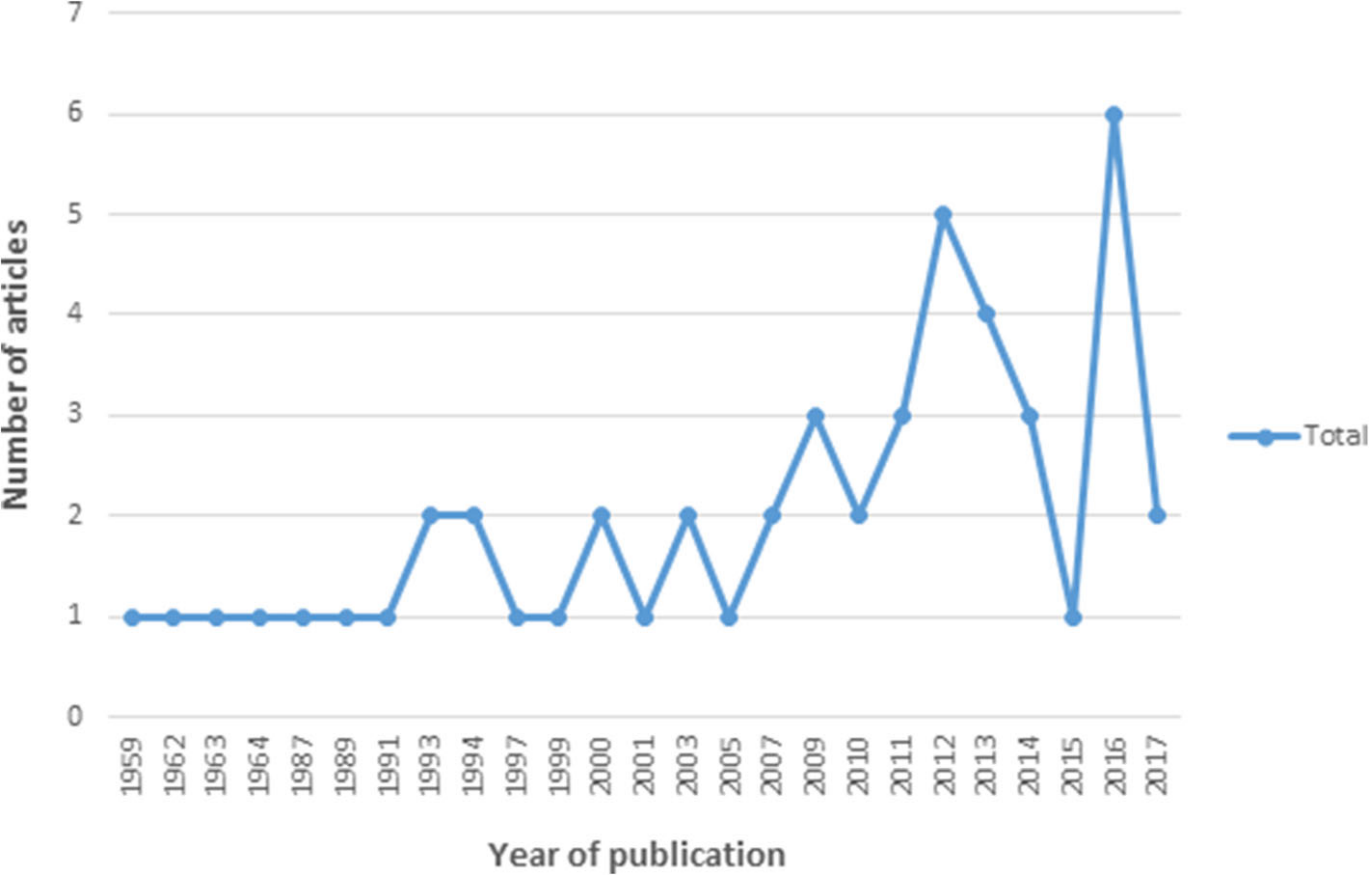
Year of publication

The majority of studies were conducted in North America (*n* = 21) and Europe (*n* = 14). Five were completed in Australasia, three in Asia and six as part of international collaborations. Publication dates ranged from 1959 to 2017 with the greatest number of articles published in 2012 and 2016 (Fig. 2).

The review included a wide range of study designs: 25 observational studies, four clinical assessment protocols, three narrative reviews, two descriptive studies, two comparisons of clinical data against machine learning algorithms, two qualitative interview studies, two cohort studies and one of each of the following: randomized controlled trial, case report, comparison of inter-rater reliability, expert consensus, quantitative survey data, chart reviews, tool validation, literature review and a systematic review. Experimental studies included in the review are described by setting and number of participants in Table 1.

**Table 1:** Study settings and number of participants (where reported)

Setting	Number of studies	Number of participants
Acute hospital	15	9086
Intermediate care	2	10,901
Community-based	6	25,322
Care homes	7	185,591
Community versus hospital rehabilitation	1	302
Day hospital	2	248
**Total**	**33**	**231,450**

The studies identified in this review included a wide range of participants, patient groups and diagnoses. In studies which considered how healthcare practitioners assessed rehabilitation potential, assessments were carried out by a single profession or as part of a multidisciplinary team (MDT) (Table 2).

**Table 2:** Healthcare practitioners involved in rehabilitation potential studies

Study	Participants	Sample size
Cunningham et al. [17]	Occupational therapist, physiotherapist, nurse, doctor	4
Hoenig et al. [18]	Physician	98
Jette et al. [19]	Occupational therapist, physiotherapist	9
McPhail et al. [20]	Physiotherapist	23
Myers et al. [21]	Nurse	unclear

The majority of studies included patients with diverse diagnoses and characteristics who were in receipt of rehabilitation assessments or interventions. Study populations were described in different ways with variables including: frailty, multimorbidity, cognitive status, functional abilities and activities of daily living. Diagnoses commonly identified included: Alzheimer’s diseases and other dementias, orthopaedic diagnoses (osteoarthritis and falls), cardiac and respiratory conditions, stroke and hip fractures. Where reported, mean ages ranged from 65 to 88.1 years (Supplementary data file three). Fourteen articles did not report on specific patient populations or conditions [18, 19, 22–33].

Findings coalesced around specific themes which are presented in Table 3:

**Table 3:** themes

Theme	Description
Definitions of rehabilitation potential	Describes how rehabilitation potential was conceptualised, either as a prognostic or retrospective measure
Who was involved in assessments	Describes who was involved in assessments and decision-making relating to rehabilitation potential
Where assessments tool place	Outlines which settings and contexts rehabilitation potential assessments took place in
When assessments took place	When in patients’ recovery trajectories rehabilitation potential assessments took place
The use of formal decision-making frameworks	Outlines how decision making frameworks such as safety checklists, prediction tools and clinical assessment protocols were applied
Components of a rehabilitation potential assessment	Describes the key domains included in rehabilitation potential assessments including: diagnoses and medication, functional abilities, mental health, social and environmental factors
How rehabilitation potential was measured	This theme explored how rehabilitation potential was measured, depending on understanding rehabilitation potential as a prognostic or retrospective measure
External factors influencing the assessment of rehabilitation potential	Describes factors such as training, skills, experience and availability of rehabilitation resources required to deliver rehabilitation programmes
Markers of success	Describes optimum outcomes of rehabilitation programmes in terms of improvement, maintenance or managing declining abilities and function

### Definitions of rehabilitation potential

Definitions demonstrated considerable heterogeneity and a lack of consensus. The term was used prognostically to describe an individual’s potential for restoration of function [28, 34, 35] or predicted benefit from MDT rehabilitation [36]. Cunningham, Mosqueda and New [17, 27, 29] adopted the definition provided by Rentz:

“*The prognostic indicator of how the patient will perform within a standard inpatient rehabilitation program … involving an estimation of the patient’s personal strengths (i.e., level of motivation/cooperation, cognitive status and personality constellation), medical complications and familial support as they interface with therapies and rehabilitation environment … estimates the individual’s capability of cooperating with a rehabilitation program and making measured functional gains in ambulation and self- care … appraising whether the patient’s current quality of life can be improved upon despite chronic or multiple disabilities.”* [6].

A number of authors [18, 26, 37–42] adopted a functionally-orientated approach to definitions where individuals had rehabilitation potential if they were likely to achieve restoration of function after an acute event. Hoenig et al. [18] considered that rehabilitation potential was better expressed by gaining improvements in quality of life rather than by functional gain alone. Gray et al. [24] and Hartley et al. [41] used place of residence as a proxy for functional ability whereby individuals had rehabilitation potential if they were predicted to be likely to be discharged back to their usual place of residence after an acute episode of ill health.

In contrast, rehabilitation potential was defined as being present if the individual undergoing rehabilitation and/or a member of the continuing care team thought the individual was capable of increased independence in some objectively measured functional areas [3, 43–46]. This definition was further refined by Zhu et al. [4, 47] whereby true rehabilitation potential was said to be present if an individual demonstrated measurable improvements in ADL functioning (measured using the interRAI ADL long form) over a period of one year or if they remained at home at the end of the rehabilitation intervention.

In three studies by Johansen et al. [48–50] a working definition developed by the Norwegian Government, was adopted which described rehabilitation potential as the “*physiological and psychological possibilities of a disabled person to restore, improve on maintain an optimal level of function and quality of life”* [51]. Whilst this definition emphasises the relationship between physical and psychological health and well-being, it was not specific to older people living with frailty.

Three studies were identified which stated that they selected patients for rehabilitation on the basis that they had rehabilitation potential [52–54] but robust operational definitions were not given. Badriah et al. [53], designed a retrospective measurement of rehabilitation potential based on the Functional Independence Measure (FIM), where rehabilitation potential was calculated by dividing the change in FIM total score at the beginning of rehabilitation therapy and hospital discharge by the FIM total score target (total maximum FIM minus FIM score at the start of rehabilitation). Rehabilitation potential was assumed to represent an improvement in functional abilities.

### Who was involved in the assessment of rehabilitation potential

Rehabilitation potential assessments were completed by: physicians [27, 34, 40], rehabilitation nurses [21], untrained home care staff [3, 38], disability or medical assessors [24, 25, 28, 32, 33] or an MDT [17, 29]. It was unclear from all studies how assessments guided decision-making and who made the final decision about rehabilitation potential. Patients or clients and carers were included in rehabilitation assessments [24, 25, 30, 37, 44] but the extent of their involvement or influence on decision-making was unclear. Chang et al. [3] assessed the differences between self-perceived and carer-evaluated rehabilitation potential among care home residents in Taiwan. The study reported that 63.2% (*n* = 367) of residents believed that their physical function would improve, but just 9.8% (*n* = 57) of their caregivers deemed them to have rehabilitation potential.

### Where rehabilitation potential was assessed

Assessments took place in outpatient geriatric clinics [28, 55], intermediate care units [48, 52], acute or subacute geriatric inpatient wards [32, 33, 41, 56, 57], inpatient rehabilitation units [17, 20, 28, 29, 36, 37, 53, 58–61], care homes [3, 28, 34, 35, 38, 40, 43, 44, 62], rehabilitation situated in care homes [39] and day hospitals [42, 54]. Some studies included multiple sites where rehabilitation took place in either the patient’s own home, inpatient setting or nursing homes [46, 48, 49]. In some studies it was unclear where the assessment of, or decision about, an individual’s rehabilitation potential took place [4, 18, 26, 27, 45, 47, 63].

### When assessments of rehabilitation potential were completed

In studies which specifically explored rehabilitation potential, the decision that an individual did or did not have rehabilitation potential was predominantly made at a single time point. Assessments occurred at the time of deciding on patient suitability for admission to a rehabilitation unit [40], to guide care planning after a hospital admission [3, 17, 29], as a snapshot for a study [34] or during application for state benefits [28]. Some studies used multiple time point assessments: at admission and discharge from rehabilitation services [21] and at baseline and one-year follow up [35]. In other studies it was unclear when the assessment and decision was made [4, 27, 38, 47]. Some tools sought to assess individuals’ pre-morbid abilities in the hours or days leading up to a hospital admission [24, 25].

### The use of formal decision-making frameworks

The identification of an individual’s rehabilitation potential was said to involve clinical judgement and reasoning [17, 21, 41], but there was limited evidence for the use of formal decision-making frameworks. In one study a Pre-Admission Screening checklist [58] was developed from a sample of 549 referrals over a six month period with medical charts reviewed for risk factors for readmission to acute care from a rehabilitation unit. A type of safety checklist was developed to guide decision making but was found to be largely subjective and unsubstantiated. Clinicians were asked to use a simple binary rating of yes, no or not applicable on absolute and relative contraindications to rehabilitation and on patients’ levels of motivation, and ability to tolerate and participate in rehabilitation.

Jupp et al. [59] developed a tool to aid clinicians in predicting outcomes after acute hospitalization and guide rehabilitation assessments. It was based on factors linked to discharges to residential or nursing home placements. The tool incorporated assessments of gait, eyesight, mental state and sedation (GEMS). In the validation study, patients admitted to care homes were found more likely to have abnormal vision, impaired cognitive abilities, gait abnormalities and taking sedative medications.

The interRAI ADL and IADL Clinical Assessment Protocols (CAP), developed for acute and community-dwelling populations [24, 25, 32, 33], provided decision-making frameworks for use in older and vulnerable populations. An overall score indicated whether the individual ‘triggered’ to prevent decline, facilitate improvement or triggered no action. A series of clinical prompts and care plans were then recommended to guide care planning. Two studies by Zhu et al. [4, 47] compared the use of CAPs with a computer algorithm to guide rehabilitation potential decision making in the Canadian home care setting. Findings indicated that both the *K-*nearest neighbour algorithm [4] and Support Vector Machine [47] had superior predictive powers for calculating rehabilitation potential and subsequent rehabilitation outcomes when compared to the ADLCAP. Further work to refine and operationalise these tools is required to understand the practical implications of applying big data to clinical decision-making.

### Components of a rehabilitation potential assessment

Two studies recommended that holistic assessments were required which addressed biopsychosocial needs and abilities of patients [29, 45]. However, there was a lack of detail about the composition of these assessments. Key areas that were identified included: diagnoses and medication, functional abilities, mental health, social and environmental factors.

#### Diagnoses and medication

The evidence suggested a pertinent role for assessing co-morbidities and diagnoses [4, 18, 19, 21, 23, 28–33, 39–41, 46–50, 52, 54, 55, 58–62] which were likely to affect rehabilitation participation or outcome. These were typically measured by counting the type and number of underlying diseases [26, 39] or using the Charlson Co-morbidity Index [64]. Medical stability was frequently seen as a prerequisite for an individual being able to take part in or tolerate rehabilitation [29]. Common features of assessments included the identification of medications which may affect rehabilitation outcome or participation [24, 25, 32, 33, 39], nutritional status [24, 25, 32, 33, 39, 60], pain [21, 24, 25, 30, 32, 33, 61], continence [17, 25, 26, 39, 61], tissue viability [62] and communication including vision and hearing [24, 25, 30, 32, 33, 39, 46]. There was a lack of evidence to support the exact composition of medical components of rehabilitation potential assessments.

#### Functional ability

Assessing and identifying functional abilities was strongly represented in the data. They were largely assessed and understood through assessing ADLs [4, 19–21, 23–25, 27, 30, 32, 33, 35–39, 41, 42, 44, 46, 48–50, 53, 54, 59–61, 63, 64]. Some studies were more specific with their definitions of function such as mobility [4, 21, 47, 56, 61], transfers [58], or occupational abilities [28]. Specific issues such as muscle strength neurological deficits or sensation [26, 34, 39] were included. Assessment of IADLs describing key life tasks such as managing finances, cleaning, shopping and meal preparation were identified [24, 25, 33, 61]. Impairments in IADL can often be present in those with mild cognitive deficits and the early stages of dementias [65] so may be an important indicator of cognitive abilities and function.

#### Mental health and psychological abilities

Establishing an individual’s psychological abilities or deficits was frequently included in rehabilitation potential assessments [20, 30, 31, 41, 49, 50, 60]. Studies cited that they specifically considered individuals cognitive abilities [4, 18, 20, 21, 23–25, 32, 33, 39, 42, 44, 46, 49, 50, 53, 60]. Gray et al. [32] stated that assessing cognitive skills for decision-making was essential, specifically short term memory recall, procedural and situational memory. An assessment of motivation [27], mood [24, 25, 32, 37, 39, 46], disruptive behaviours [21, 24, 25, 29, 30, 39, 40, 46] and depression [23, 24, 30, 39, 46] were also found to be included. Motivation was described as being present if the patient was eager to participate in therapy and took responsibility for being actively involved in their self-care [27]. The Kemp model of motivation [66] was proposed, taking into account patient wants, beliefs and rewards, offset by the costs of participating in the rehabilitation programme.

#### Social

An assessment of rehabilitation potential was found to require an understanding of an individual’s social circumstances [18, 21, 22, 35, 37, 39, 46, 52]. Understanding social status and conditions were important factors in determining the recovery of older community dwelling adults who received intermediate care rehabilitation following an acute hospital admission [52] where the ability to live at home was reported to be a *“good and practical measure of recovery”.* Social situation, where an individual lives and the type of support they received were found to be strong predictors of rehabilitation outcome [63]. Mosqueda [27] outlined that understanding the reliability and number of existing social support mechanisms were essential components of rehabilitation potential assessments. Caradoc-Davies et al. [37] explored the perceived benefits of rehabilitation between health professionals and clients, finding that those with strong social support mechanisms were more positive about the potential benefits of rehabilitation.

#### Environmental

The literature highlighted the need to assess an individual’s environment [27–30, 54]. Mosqueda [27] suggested that environmental assessments should include understanding the environment of the usual place of residence and the current or proposed rehabilitation venue. This view was supported by Nagi [28], who stated that the environment should be considered in terms of the individuals’ level of functioning within that specific environment, suggesting that assessments were context-specific.

### How rehabilitation potential was measured

A number of measures were identified in studies specific to rehabilitation potential. Chang et al. [3] found significant disagreement between residents and caregivers on whether they thought rehabilitation would improve a residents ADL’s. Myers et al. [21] found a significant relationship between nurses assessment of rehabilitation potential at admission and functional status as measured through ADLs at discharge (r = 0.20, R^2^ = 0.04, *P* < 0.001). Cunningham et al. [17] proposed a binary response where members of the MDT were asked to rate the rehabilitation potential of 27 consecutive patients admitted onto a geriatric rehabilitation ward as either good or poor. They found that agreement between professionals was poor (kappa = 0.21).

Other studies adopted retrospective measures, comparing outcome measures before and after rehabilitation programmes. Measures adopted included changes in individuals ADL functioning [4, 47] where gains were seen as a positive affirmation of rehabilitation potential.

New [29] developed a traffic light system to classify a patient’s appropriateness for rehabilitation and by proxy their rehabilitation potential. This model, developed by expert opinion, proposed that ‘green light’ patients were always appropriate for rehabilitation, those with conversion and personality disorders, obesity or specialist nursing needs were classified as ‘orange’ (proceed with caution) and for patients with limited life expectancy, lack of capacity and severe dementia as red and not appropriate for rehabilitation. This system was not however designed specifically for older people, rather for a heterogeneous inpatient population.

Most aspects of medical interventions were not measured or categorized in a way that could be easily reported. Those that were quantifiable were largely measures of frailty or symptom scores.

Morghen et al. [60] was the only study which sought to measure and evaluate the impact that patient participation had on predicting rehabilitation gains or outcomes. They found that participation was independently associated with functional gain in an older people’s inpatient rehabilitation setting. Participation was assessed using the Pittsburgh Rehabilitation Participation Scale (PRPS) [67], and functional gain was measured using the Montebello Rehabilitation Factor Score [68]. The PRPS measures participation during therapy sessions, where clients were rated using a Likert Scale of 1–6 (1 = refusal to participate in a session and 6 = excellent participation in all exercises, taking an active interest in exercise and/or future therapy sessions). Moseley et al. [26] and Wells et al. [31] proposed the Goal Attainment Scale to measure rehabilitation outcomes, whereby patient-centred goals are set and percentage attainment was measured.

### External factors influencing the assessment of rehabilitation potential

Staff skills, training and experience were found to affect the transaction of rehabilitation potential assessments [26, 29, 38, 64]. Two separate concepts emerged from the literature: the skills of staff to assess rehabilitation potential and skills for providing rehabilitation interventions. Fortinsky [38] proposed that training and clinical judgement were key factors, stating that older adults with complex needs may never reach the ideal of maximised function due to clinical judgements and policy guidelines that carry vague and conflicting messages about rehabilitation potential. Moseley [26] and Mofina and Guthrie [45] suggested that staff needed a thorough understanding of referral criteria to rehabilitation services in order to decide on the suitability of individuals to rehabilitation.

Funding and availability of resources were considered in rehabilitation potential assessments. Mosqueda [27] stated that economic reality influenced rehabilitation potential, whereby resources are limited or rationed through government commissioning or insurance. Although an individual may demonstrate gains from rehabilitation during their inpatient stay, if resources are not available to continue programmes of rehabilitation, gains may not be maintained and benefit may therefore not be realised. In this context, Gordon [40] found that staff over-estimated an individual’s rehabilitation potential for fear of omission; in other words, they offered rehabilitation even if they were unsure of the benefit. This ethical dilemma is further supported by New [29] who highlighted the tensions clinicians faced in allocating resources including the potential for injustice and bias, utility and beneficence and how these factors may influence the decision that an individual does or does not have rehabilitation potential.

### Markers of success

The majority of studies identified in this review included patients who had been deemed to have rehabilitation potential, rather than studies which explored or tested the assessment of rehabilitation potential. As a consequence, a successful outcome of rehabilitation potential was frequently linked to rehabilitation outcomes specific to the study design, aims and objectives.

Frequently, improvement was identified as the optimum outcome associated with rehabilitation or rehabilitation potential amongst older people. Improvement was described as a return to premorbid abilities or an improvement in function [3, 4, 17, 18, 24–26, 32–34, 37, 40, 41, 45–50, 52, 53, 58, 60, 64]. However, some studies recognised that improvement may not always be feasible in this population. Muller et al., Gray et al. and Fusco et al. [24, 35, 39] stated that maintaining an individual’s current status and abilities was also a successful outcome. Poulos et al. [30] further embraced this notion, proposing that reablement programmes in dementia should go beyond improvement and consider maintenance and managing or delaying declining abilities. Assessments should identify and address causes of functional decline discrete from the natural progression of the underlying dementia diagnosis, such as medication management, acute or comorbid medical conditions, deconditioning or lack of activity.

## Discussion

This study found considerable variations in definitions of rehabilitation potential and in some cases, an absence of definition. Rehabilitation potential was found to encompass two different, but inter-related, concepts of prognostication and outcome measurement. Prognostic rehabilitation potential described the prediction of what could be achieved through rehabilitation, whereas outcome-based rehabilitation potential considered what had been achieved. The locations of rehabilitation potential assessments were highly contextualized by the study designs and aims.

Prognosis involves the prediction of the future course and outcome of disease processes concerning either their natural course or outcome after treatment [69]. Prognostic methods in medical and rehabilitation decision-making allow for wider contextual factors to be taken into account [70]. These factors are commonly affected by frailty, old age and multi-morbidity. Single conditions and diagnoses are more predictable in terms of their trajectories and response to treatment, however, multi-morbidity, frailty and acute ill health make for a very unpredictable rehabilitation context [71]. In contemporary clinical practice, older people living with frailty frequently present with acute illnesses superimposed on underlying conditions and physiological decline. This presents clinicians with particular challenges in understanding and predicting recovery [72, 73] and challenges researchers in establishing the effectiveness of rehabilitation interventions.

Rehabilitation potential was also found to be used as a proxy for entry criteria into rehabilitation studies. In that patients deemed not to have rehabilitation potential were excluded from studies based on the belief that they would not benefit or respond to rehabilitation interventions. This frequently included those with moderate to severe levels of cognitive impairment [19, 52, 64] who are regular recipients of in-patient hospital care. If the evidence base for frailty rehabilitation is to progress, patients with cognitive impairment must be recruited to studies so that their true rehabilitation potential can be understood. There have been suggestions that the term rehabilitation potential may lead to rationing of services particularly in older adults with cognitive impairments [9]. Age based rationing of services presents significant practical and ethical challenges in terms of allocation of services and resources and the term rehabilitation potential may further reinforce outdated notions of rehabilitation benefit [11].

Rehabilitation potential was used as a measure of rehabilitation outcome, in that individuals ‘had rehabilitation potential’ if they achieved favourable outcomes. Based on retrospective analysis, these variables help inform clinicians’ predictions of what an individual may be capable of, but in isolation they do not capture the complexity of human behaviour and nuances of frailty and multimorbidity. However, Enderby et al. [8] warn that variables which are strong predictors may hide the subtleties associated with an individual’s recovery and clinical decision-making.

Unsurprisingly, domains relevant to the World Health Organization’s International Classification of Functioning, Disability, and Health (WHO ICF) [16] featured prominently in the findings of this mapping review. This may be explained by the use of the ICF in the a priori analytical framework but is also indicative of the impact that the ICF has had on contemporary clinical practice. This study has demonstrated that knowledge of physical attributes and underlying diseases and conditions are integral to assessments of rehabilitation potential. Findings from this study draw many parallels with Comprehensive Geriatric Assessment (CGA) models of care which seek to provide an iterative approach of assessment and case management focus on medical, mental health, functional capacity, environmental and social circumstances [74, 75]. CGA aims to place patient and carers needs at the centre of the relationship through the use of targeted goal setting which enables interventions, such as rehabilitation to be identified, delivered and revewied. This review identified evidence to support the assessment of medical, mental health and functional abilities in terms of rehabilitation potential but limited evidence to focus on environment and social circumstances. Recent literature has suggested that spirituality and economic status should also be considered for a truly holistic assessment [76], but no supporting evidence for the inclusion of these domains in an assessment of rehabilitation potential was identified in this review.

Personal and participatory factors are part of the ICF [16] and this study found that motivation and participation played a key role in assessments of rehabilitation potential. Motivation is a complex construct that has been widely explored in relation to rehabilitation in traumatic brain injury, stroke and sports medicine but less frequently amongst older people living with frailty. Siegert et al. [77] propose that exploring an individual’s motivation, emotions and goals allows for an understanding of how they will react with rehabilitation programmes, whereas prognosis or prediction considers variables and outcomes. Rehabilitation potential assessments should consider prognostic, performance and participatory approaches for maximal rehabilitation outcomes to be achieved. Commonly cited ‘barriers’ to rehabilitation such as poor cognition and low mood [78] can all have a profound impact on an individuals’ ability to be motivated to take part in and achieve beneficial outcomes from rehabilitation interventions. It remains unclear which items within these tools best correlate to or predict rehabilitation potential in older people living with frailty.

It is clear that solely focusing on the physical effects of frailty will not address the complex, highly individualised and fluctuating needs of older people living with frailty. Clinicians need to consider the wider social implications of ageing and the impact these have on continued quality of life and control over individuals lives. The inclusion of environmental and social domains of assessment identified during this review may go some way to remedy this medical and physical bias, but further evidence is needed to understand how these domains relate to rehabilitation potential. Rehabilitation potential was largely assessed at singular time points, with subsequent reviews of outcome measures completed retrospectively.

### Strengths and limitations

Due to the heterogeneous nature of frailty presentations and rehabilitation interventions a mapping review was ideally suited to map this complex field. This enabled the context and mechanism of frailty rehabilitation to be explored, essential in understanding complex interventions [79].

Of the 49 studies included in this review, 24 were from either Anglophone countries (UK, USA, Canada or America) or from European countries which have a tradition in publishing in English language journals. The predominance of studies from Anglophone countries may represent selection bias by limiting selection criteria to the English language. Asian countries have been found to publish less frequently [80], but this study included three publications from Asian countries and a total of six international collaborations.

This study excluded evidence from books and hence the most commonly cited definition of rehabilitation potential by Rentz [6]. Whilst academic books are subject to editorial review, they do not always undergo the same scrutiny as articles in peer-reviewed journals. These sources, commonly classified as grey literature, are frequently excluded from evidence appraisal methods, but can provide new insights and help contextualise research evidence [81].

It proved challenging to identify studies which solely explored rehabilitation potential in relation to frailty. This may represent a limitation in search terms or engines used, but more likely represents the lack of evidence in rehabilitation decision-making and the emerging field of frailty rehabilitation. The studies included in this review comprised a broad range of clinical conditions and patient groups. This study sought to exclude articles which included patient participants in receipt of specialist stroke, palliative and fracture services. However, many of the studies identified included patients with these diagnoses.

## Conclusion

This review identified considerable heterogeneity in definitions and use of the term rehabilitation potential and in some cases an absence of definition despite it being used as an entry criterion into a study. It was found to be poorly understood and judged differently by different people at different times. Rehabilitation potential was found to encompass two different but interrelated concepts of prognostication and outcome measurement. Limited evidence was identified which specifically considered rehabilitation potential amongst older people living with frailty. Current tools and approaches provide a snapshot of an individual’s abilities and conditions which failed to capture the dynamic nature and fluctuations associated with frailty and rehabilitation. Snapshot approaches further enhance the risk of age-based rationing of services where those who might benefit from rehabilitation are denied access to interventions. New aggregative approaches to measures and abilities over time are required which allow for the prognostication of outcomes and potential benefits of rehabilitation interventions for older people living with frailty.

## Supplementary Information

**Additional file 1.** Supplementary data file one- Justification for search terms and database selection.

**Additional file 2.** Supplementary file three- Patient participants demographics (where reported).

## Data Availability

The datasets used and analysed during the current study are available from the corresponding author on reasonable request.

## Abbreviations

ADL: Activities of Daily Living
CAP: Clinical Assessment Protocol
CGA: Comprehensive Geriatric Assessment
FIM: Functional Independence Measure
IADL: Instrumental Activities of Daily Living
MDT: Multi-disciplinary Team
WHO ICF: World Health Organization International Classification of Functioning, Disability and Health
