# Supplementary material for: Rehabilitation potential in older people living with frailty: a systematic mapping review
Source: BMC Geriatr. 2021 Oct 7;21:533. doi: 10.1186/s12877-021-02498-y (PMC8496021; doi:10.1186/s12877-021-02498-y)
Supplement: Supplementary file 1 — Additional file 1. Supplementary data file one- Justification for search terms and database selection. [file 12877_2021_2498_MOESM1_ESM.docx]

**Supplementary material**

Supplementary data file one- Justification for search terms and database selection.

| **Database** | **Search and Index Terms** | **Justification for inclusion** |
| --- | --- | --- |
| Medline (OVID 1946- present) | “Rehabilitation potential” OR Frail Elderly/ or "Aged, 80 and over"/ or Aged/ AND "Physical and Rehabilitation medicine"/ or Rehabilitation/ | Biomedical and clinical medical literature, includes multidisciplinary research |
| CINAHL Plus full text (EBSCO) | (Rehabilitation potential) OR (MH "Frail Elderly") OR (MH "Aged, Hospitalized") OR (MH "Aged, 80 and Over") AND (MH "Rehabilitation) | Nursing and Allied Health Professionals journals reflecting the multidisciplinary nature of rehabilitation practice and rehabilitation potential assessments |
| EMBASE (Ovid) | Rehabilitation potential/OR frail Elderly/OR aged/Or (aged 80 and over) AND Geriatric rehabilitation AND rehabilitation | International coverage across biomedical journals from 90 countries |
| AMED (Allied and Complementary Medicine, OVID) | Rehabilitation potential/OR Aged/OR Frail Elderly/ AND Rehabilitation/ | Research from Physiotherapy, Allied Health and nursing, reflecting MDT nature of rehabilitation. European coverage that may be excluded from CINAHL |
| **Database** | **Search and Index Terms** | **Justification for inclusion** |
| PsycINFO (OVID) | Rehabilitation potential OR Aging AE OR Geriatric patients AE OR Geriatrics AE AND Rehabilitation AE | Focuses on behavioural and social sciences, embracing the holistic approach of rehabilitation |
| PEDRO | Frailty aged rehabilitation assessment | Physiotherapy evidence database of randomized trials, systematic reviews and clinical practice guidelines |
| Cochrane Library | “Rehabilitation potential” OR “older people” OR “rehabilitation.” | Focus on medicine and healthcare specialities to inform healthcare, decision-making and clinical practice |
| Web of Science | TOPIC" older people" and TOPIC "rehabilitation assessment" OR “Rehabilitation potential” | Includes citations from across healthcare |
| ProQuest Dissertations and Theses | “Older people” OR “frail elderly” AND “Rehabilitation” OR “Rehabilitation potential” | Searching across worldwide databases of dissertations and theses for unpublished work |
| **Database** | **Search and Index Terms** | **Justification for inclusion** |
| Trip (Turning Research into Practice) | “Older people” AND “rehabilitation” OR “rehabilitation potential” | Focus on evidence which explores research into clinical practice |
| EThOS | “Older people OR frail elderly AND rehabilitation” OR “rehabilitation potential” | UK thesis repository |

Supplementary data file two- data extraction form

Authors

| Authors | | Year | |
| --- | --- | --- | --- |
| Title | | | |
| Journal | | | Reviewer |
| Study Setting | Country | | |
| Study Type | Sample size | | |
| Patient Group | | | |
| Participants | | | |
| Analysis | | | |
| Results/findings | | | |
| Definition of rehabilitation potential or similar descriptor | | | |
| Clinical tools used to assess rehabilitation potential | | | |
| Domains assessed | | | |
| Who completed assessment? | | | |
| When and where was assessment completed? | | | |
| What outcome measures were used? | | | |
| What decision making tools were used? | | | |
| Measures of success | | | |
| Factors influencing rehabilitation potential assessment | | | |
| Comments on quality and biases | | | |

**Supplementary data file three**

Attached as separate file
