# Supplementary material for: Rehabilitation potential in older people living with frailty: a systematic mapping review
Source: BMC Geriatr. 2021 Oct 7;21:533. doi: 10.1186/s12877-021-02498-y (PMC8496021; doi:10.1186/s12877-021-02498-y)
Supplement: Supplementary file 2 — Additional file 2. Supplementary file three- Patient participants demographics (where reported). [file 12877_2021_2498_MOESM2_ESM.docx]

Supplementary file three- Patient participants demographics (where reported).

| **Author** | **Patient group** | **Sample size** | **Age (years, mean)** | **Frailty or Comorbidity measures** |
| --- | --- | --- | --- | --- |
| Aberg et al. (199) | Stroke, hip fracture, spinal injury, myocardial infarction, respiratory insufficiency | 15 | x | x |
| Abrahamsen et al (200) | Medical (infection), orthopaedic (fallers, contusions and fractures) | 961 (medical n=615) (orthopaedic n=346) | 85 | 567 (59%) had 5 or more diagnoses |
| Alekseyev et al. (201) | Stroke, medical debility, cardiac, neuromuscular, respiratory, other | Unclear | 67.7 | x |
| Arling et al. (202) | Mixed morbidities (cardiovascular disease, stroke, cancer, dementia, neurological, endocrine and psychiatric disorders | 558 | 77 | x |
| Arling et al. (203) | Mixed cognitive impairments, multi-morbidity | 1419 (29.2% cognitive impairment, 38.8% pain, 62.6% cardiovascular) | 82.6 | x |
| Badriah et al. (204) | Stroke, orthopaedic, disuse syndrome | 835 (stroke n=205, orthopaedic n=441, disuse n=189) | 76.49, 78.7, 79.17 | x |
| **Author** | **Patient group** | **Sample size** | **Age (years, mean)** | **Frailty or Comorbidity measures** |
| Cameron et al. (205) | Stroke, hip fracture, joint replacement, amputation, orthopaedic | 560 (stroke n=87, hip fracture n=103, joint replacement n=96, lower limb amputation n=12, other orthopaedic n=170, other rehabilitation n=92) | 80 | x |
| Caradoc-Davies et al. (206) | Unclear | 94 | x | X |
| Chang et al. (129) | Physical and cognitive impairment (diagnosis not given) | 581 (79.7% cognitive intact, 20.2% mild-moderate cognitive impairment, 92.8% physically independent, 7.2% physically dependent) | 80.9 | X |
| Chou et al. (207) | Unclear but impaired in activities of daily living | 83 | 80.9 | x |
| Cunningham et al. (134) | 15% stroke, 11% hip fracture, 26% cardiorespiratory, 7% musculoskeletal, 4% cognitively impaired, 37% other | 27 | 81 | x |
| Eagle et al. (208) | Depression, stroke, degenerative joint disease | 113 | 79.6 (treatment group) | x |
| Fortinsky (209) | In a care home, unclear diagnoses | 8665 | 60 years plus (mean not given) | x |
| **Author** | **Patient group** | **Sample size** | **Age (years, mean)** | **Frailty or Comorbidity measures** |
| Fusco et al. (210) | Stroke, hip fracture, osteoarthritis, Parkinson's disease | 598 (44.3% stroke, 31.8% hip fracture, 11% OA, 9.5% Parkinson's disease) | 82 | 2.3 +- 1.5 number comorbidities |
| Gordon et al. (127) | Stroke, mental deficiency, hip fracture, amputation, other | 112 | 76 | x |
| Haley et al. (211) | Fractures, cardiac conditions | 75 | 81.3 | Edmonton Frail Scale 8.65 mean, 6 mean comorbidities |
| Hartley et al. (212) | Cognitively intact and cognitive impairment admitted to an acute hospital | 590 | 84.6 and 88.1 | Charlson Comorbidity Index (CCI), Clinical Frailty Score for each group reported |
| Hershkovitz & Brill (213) | Stroke, hip fracture, joint replacement, other conditions | 135 | 72 male, 74 female | x |
| Johansen et al. 2011 (214) | Stroke, osteoarthritis, hip fracture, chronic diseases | 202 (Stroke n=34, Osteoarthritis n=23, Hip fracture n=82, other n=61) | 80.7 | x |
| Johansen et al. 2012 (215) | Stroke, osteoarthritis, hip fracture, chronic diseases | 255 (17% stroke, 13% osteoarthritis, 36% fracture, 34% other) | 81.7 | x |
| Johansen et al. (133) | Stroke, osteoarthritis, hip fracture, chronic diseases | 302 | 80.7 and 80.2 | x |
| **Author** | **Patient group** | **Sample size** | **Age (years, mean)** | **Frailty or Comorbidity measures** |
| Jupp et al. (216) | Multi-morbidity (not defined) | 624 | 80.9, 81.6, 85.1 | x |
| Leung et al. (217) | Stroke, hip fracture, medically complex, other musculoskeletal conditions | 104 (29.8% hip fracture, 30.7% medically complex, 18.3% stroke, 21.2% other) | 81.6 | 6.8 +- 2.5 number of comorbidities |
| McPhail et al. (186) | Orthopaedic, stroke, other neurological conditions, geriatric deconditioning, other disabling conditions requiring rehabilitation | 272 | 79 (low cognition) 71.7 (high cognition) | x |
| Mofina & Guthrie (218) | Impairments in cognition, activities of daily living and instrumental activities of daily living | 111,804 | 83.2 | CHESS scale (63% low, 37% mild or severe) |
| Morghen et al. (219) | Orthopaedic, cardiac, pulmonary, neurological, immobility syndrome | 556 | 81.6 (low gain) 78.2 (high gain) | Cumulative illness rating scale 1.7 (low gain) 1.6 (high gain) |
| Muller et al. (220) | Unclear - impaired activities of daily living | 400 | x | x |
| Myers et al. (187) | Medical and surgical diagnoses | 362 | 79.7 | x |
| **Author** | **Patient group** | **Sample size** | **Age (years, mean)** | **Frailty or Comorbidity measures** |
| Reynolds et al. (124) | Selection of diagnoses, given by system not diagnosis | 1480 | 77 | 2.4 major diagnoses per patient |
| Seematter-Bagnoud et al. (221) | Hip fracture, osteoarthritis, Cardiovascular, respiratory, cerebrovascular diseases, Gastrointestinal | 2754 | Stratified by age group (<75, 75-84, ≥ 85) | 46% had 5 or more diagnoses |
| Singh et al. (222) | Geriatric syndromes | 265 | 82.6 | By outcome using frailty index and CCI |
| Sinn et al. (223) | Co-morbidities (not defined) | 9940 (59.1% hypertension, 31.7% diabetes, 31.4% osteoarthritis, 20% Stroke, 16.9% Alzheimer's disease) | 77.9 | Adjusted for comorbidities |
| Stolee et al. (224) | Elderly rehabilitation group | 173 | 81 | x |
| Zhu et al. (225) | Unclear | 24,724 | 76.3 | 15.7% had Alzheimer's or dementia diagnosis |
| Zhu et al. (130) | Unclear | 24,724 | x | x |

X= not reported
